# Intracranial vasculature 3D printing: review of techniques and manufacturing processes to inform clinical practice

**DOI:** 10.1186/s41205-020-00071-8

**Published:** 2020-08-06

**Authors:** Petrice M. Cogswell, Matthew A. Rischall, Amy E. Alexander, Hunter J. Dickens, Giuseppe Lanzino, Jonathan M. Morris

**Affiliations:** 1 Department of Radiology, Mayo Clinic, 200 First St SW, Rochester, MN 55905 USA; 2 Suburban Imaging, 4801 West 81st Street, Suite 108, Bloomington, MN 55437 USA; 3 Department of Neurosurgery, Mayo Clinic, 200 First St SW, Rochester, MN 55905 USA

**Keywords:** 3D printing, Cerebral angiography, Intracranial vasculature, Patient specific models

## Abstract

**Background:**

In recent years, three-dimensional (3D) printing has been increasingly applied to the intracranial vasculature for patient-specific surgical planning, training, education, and research. Unfortunately, though, much of the prior literature regarding 3D printing has focused on the end-product and not the process. In addition, for 3D printing/manufacturing to occur on a large scale, challenges and bottlenecks specific to each modeled anatomy must be overcome.

**Main body:**

In this review article, limitations and considerations of each 3D printing processing step, as they relate to printing individual intracranial vasculature models and providing an active clinical service for a quaternary care center, are discussed. Relevant advantages and disadvantages of the available acquisition techniques (computed tomography, magnetic resonance, and digital subtraction angiography) are reviewed. Specific steps in segmentation, processing, and creation of a printable file may impede the workflow or degrade the fidelity of the printed model and are, therefore, given added attention. The various available printing techniques are compared with respect to printing the intracranial vasculature. Finally, applications are discussed, and a variety of example models are shown.

**Conclusion:**

In this review we provide insight into the manufacturing of 3D models of the intracranial vasculature that may facilitate incorporation into or improve utility of 3D vascular models in clinical practice.

## Background

Over the past several years, three-dimensional (3D) printing has markedly increased in prevalence [1] and has been applied to the intracranial vasculature for various applications, including patient-specific models, training and education, and studying hemodynamics [2–6]. The modeled pathologies include intracranial aneurysms, vascular malformations, stenosis, and vessels as they relate to a tumor [2–4, 7, 8]. As such, patients, trainees, and providers, including neuro-interventionalists, neurosurgeons, and neurologists, benefit from the use of such models.

As 3D printing becomes more prevalent, there is increased demand for its use in education, training, and operative planning. The increasing demand for 3D models necessitates efficiency in the production process, particularly at institutions in which 3D printing has become an active clinical service. Unfortunately, though, much of the prior literature regarding 3D printing has focused on the end-product and not the process. In addition, for 3D printing/manufacturing to occur on a large scale, challenges and bottlenecks specific to each modeled anatomy must be overcome. The purpose of this work is to discuss limitations and considerations of each step in the process of 3D printing of the intracranial vasculature as it relates to providing an active clinical service for a quaternary care center. In reviewing these processes, we provide insight into the manufacturing of 3D models of the intracranial vasculature that may facilitate incorporation into or improve utility of 3D vascular models in clinical practice.

## Main text

### Overview of the 3D printing process

**Fig. 1:**
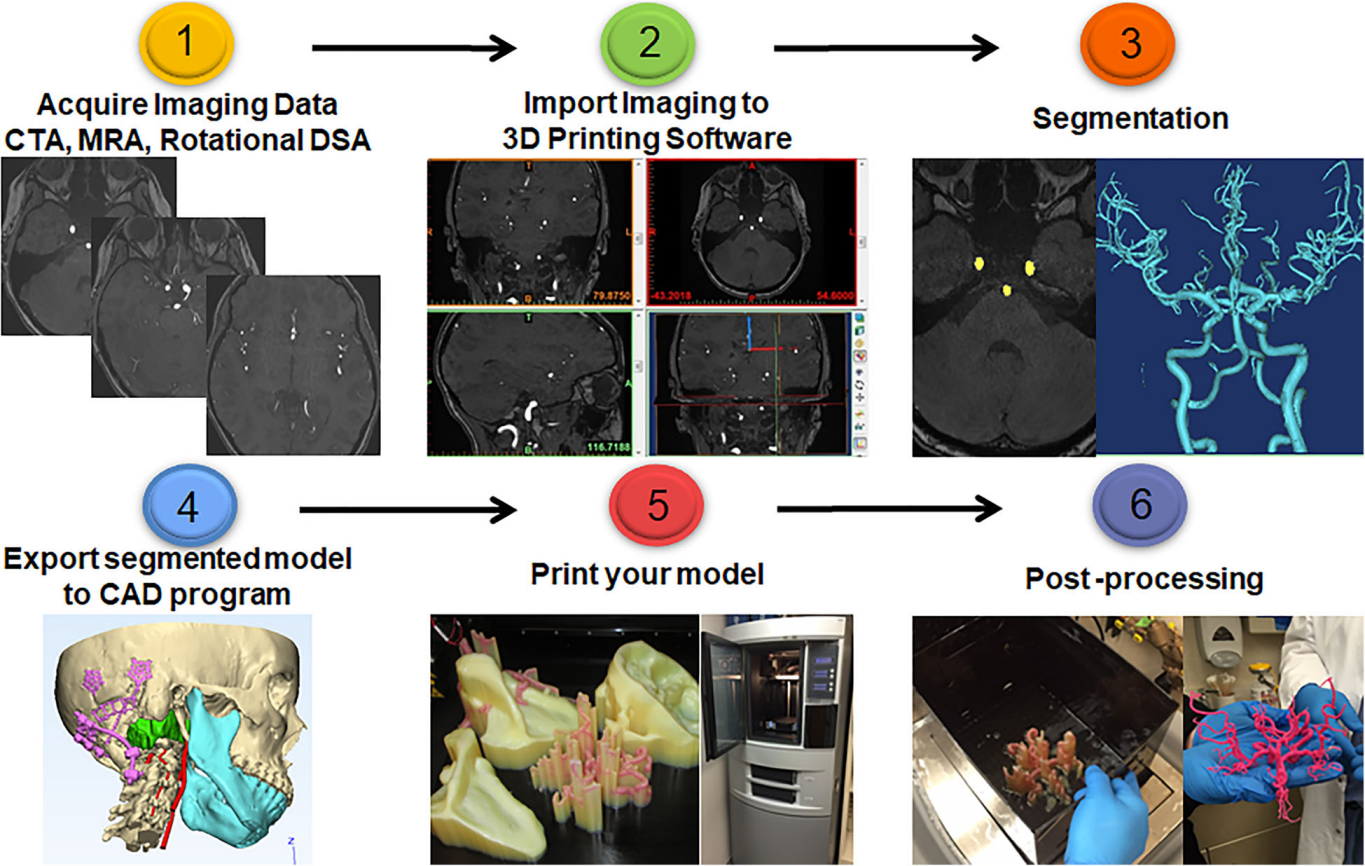
Overview of the 3D printing process. (1) Acquire angiographic data using an imaging protocol designed with the intent on 3D printing. (2) Import the imaging to 3D printing software such as Materialise: Mimics/3-Matic, 3D systems: Geomagic, OSIRIX, Toshiba: Vital Images, Autodesk, Freeware: 3D slicer, Sketchup, Blender, and many more. (3) Segment or separate the parts of the anatomy you want to print (intracranial vasculature) from the rest of the anatomy. (4) Export the STL file or segmented 3D model to the CAD program. In this step parts needed for stability must be designed, such as cylinders to hold the vertebral arteries to the skull. Surgical osteotomies maybe planned as shown in pink. (5) Print your model using your choice of print technology and material based on the need for colors, model flexibility, and support material. (6.) Post-process the model, including removal support material as shown in this example

A diagram of the 3D printing process is shown in Fig. 1. After an application is determined for a 3D model and an order is placed if in the setting of a clinical service, the 3D printing process includes the following steps: (i) image acquisition, (ii) import into 3D printing software, (iii) segmentation, (iv) processing and creation of a printable file, (v) printing, and (vi) post-processing [5, 9]. Image acquisition techniques that may be used for modeling of the intracranial vasculature include computed tomography angiography (CTA), magnetic resonance angiography (MRA), and digital subtraction angiography (DSA). The Digital Imaging and Communications in Medicine (DICOM) data is imported into 3D printing software for segmentation, which separates the anatomy you wish to print from surrounding structures. The segmented model is exported to Computer-Aided Design (CAD) software, which converts the model to a triangular mesh that is further processed for 3D printing. Various 3D printing techniques are available, and ideally the user can select which is best for a given application based on type of material properties desired. After printing the model, post-processing may include removal of the support material, smoothing, clear coating, or painting. Considerations in each of these steps as they relate to 3D printing of the intracranial vasculature are discussed below.

### Image acquisition

The image acquisition step is often overlooked as publications describe the 3D-printed end product and do not focus on optimizing the image acquisition using rotational DSA, MRA, or CTA (Table 1). If it is known a priori that a 3D model will be created from a dataset, each of these acquisition techniques may be optimized as such.

**Table 1:**
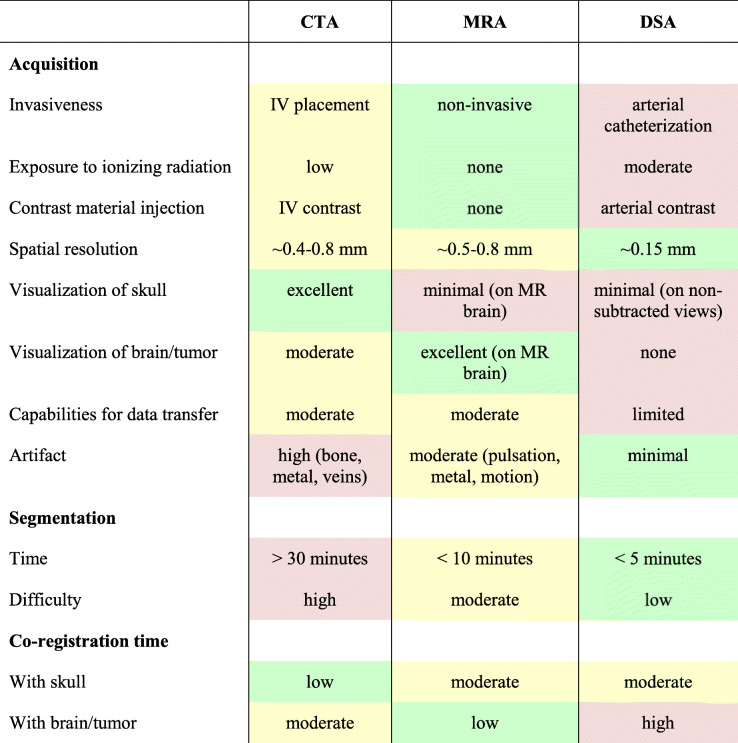
Summary of advantage and disadvantages of angiographic acquisition techniques for 3D printing of the intracranial vasculature

Shading: favorable (green), equivocal (yellow), less favorable (purple). Computed tomography angiography (CTA). Magnetic resonance angiography (MRA). Digital subtraction angiography (DSA). Intravenous (IV)

#### Digital subtraction angiography

Arterial access with selective catheterization for imaging of the internal carotid and vertebral arteries is required for DSA. Bi-plane x-ray images are rapidly acquired following administration of iodinated contrast material [10]. Benefits of DSA are very high spatial resolution with relatively few artifacts, and preserved relationship with the skull. Limitations of DSA are that it is invasive, often not part of a routine clinical workup, and requires multiple injections to image the entire Circle-of-Willis (COW). Superposition of structures on 2D DSA has been overcome with the development of 3D rotational angiography (3DRA) [11], in which images are acquired approximately every 2° over a 180° rotation to produce 3D reconstructions. A disadvantage of this technique is that each vendor has proprietary software that reconstructs the 3D images from the raw data, and vendor-provided workflows to export the reconstructed data for use in 3D printing must be obtained. Our experience has been that some vendors are hesitant to allow access to the 3D data sets for exporting specifically for 3D printing. Additionally, as mentioned above, separate acquisitions of the internal carotid and vertebral arteries are performed for an optimal exam, and each acquisition must be co-registered and segmented separately to print the entire COW.

#### Magnetic resonance angiography

Either non-contrast or contrast-enhanced methods may be used to perform MRA [12, 13]. Contrast-enhanced MRA of the intracranial vasculature is generally not well-suited for 3D printing due to unavoidable venous enhancement, obscuring segmentation of the arteries. Among the non-contrast MRA methods, 3D time-of-flight (TOF) is preferred over phase contrast, as it can provide higher spatial resolution arterial imaging without venous overlap. Additionally, TOF MRA provides high-contrast resolution, and therefore, increased ease in segmentation relative to CTA. Moreover, all intracranial vessels are acquired with one acquisition, as opposed to DSA. Limitations of TOF MRA are decreased spatial resolution compared to DSA, exaggeration of stenosis, and artifacts (pulsation, turbulent flow, metal susceptibility, and motion) [13]. Although surrounding structures, such as normal brain or tumor, are not visualized on the MRA, they may be optimally imaged with MR brain performed in the same imaging session, and therefore, easily co-registered. When performing 3D TOF MRA for 3D printing, acquisition should be performed at 3 T vs 1.5 T for improved signal-to-noise ratio. High spatial resolution is necessary; slice thickness of 1.0–1.5 mm and in-plane resolution of 0.5–0.8 mm or better is recommended.

#### Computed tomography angiography

Computed tomography angiography is performed following administration of iodinated contrast material with timing of the acquisition to the peak arterial phase using a bolus tracker or test bolus technique [14]. Benefits of CTA include wide availability, speed (and therefore, less motion artifact), high spatial resolution, large field-of-view, and the ability to simultaneously image bone and soft tissue. Limitations of CTA include radiation, use of intravenous (IV) contrast, improper bolus timing/venous contamination, metal streak artifact, and overall difficulty in segmentation due to normal structures with signal similar to that of the arteries. For printing a 3D model, the acquisition is ideally performed using a multidetector helical acquisition with isotropic spatial resolution and voxel size of 1mm^3^ or smaller. Data from the CT may be reconstructed using different filters or algorithms (bone vs soft tissue), and the soft tissue reconstruction is preferred for segmentation, as it is less noisy. Iterative metal artifact reduction methods may be applied to reduce streak artifact in patients with hardware [15]. Dual energy CT [16] may also be implemented to reduce metal artifact, as well as aid in separation of bone and contrast-enhanced vessels.

### Import imaging to 3D printing software

Following acquisition the DICOM datasets must be transferred to the 3D printing software from the clinical machine. For 3D printing to become an efficient clinical service, an institutional process for transferring the data from the scanners must be created and linked to an order within the electronic medical record [1]. One solution is to create a terminal that each scanner may send 3D printing data to or may be used to pull the data from the clinical storage system. Of note, the entire exam should not be imported to the 3D printing software due to data storage limitations. Rather, only the relevant series from each acquisition should be imported for segmentation.

### Segmentation

Segmentation involves utilizing several tools to separate the anatomy you wish to print, in this case the intracranial vasculature, from the surrounding structures. Many automated and manual methods for segmentation are available and have been discussed in the literature [17]. Commonly used methods include thresholding, which separates anatomy based on signal intensity or Hounsfield units, and region growing, which keeps only the pixels that are connected to the chosen target structure or volume. Segmentation is one of the greatest bottlenecks in the process of 3D printing the intracranial vasculature, particularly if the images were not acquired using an ideal technique with anticipation of 3D printing.

**Fig. 2:**
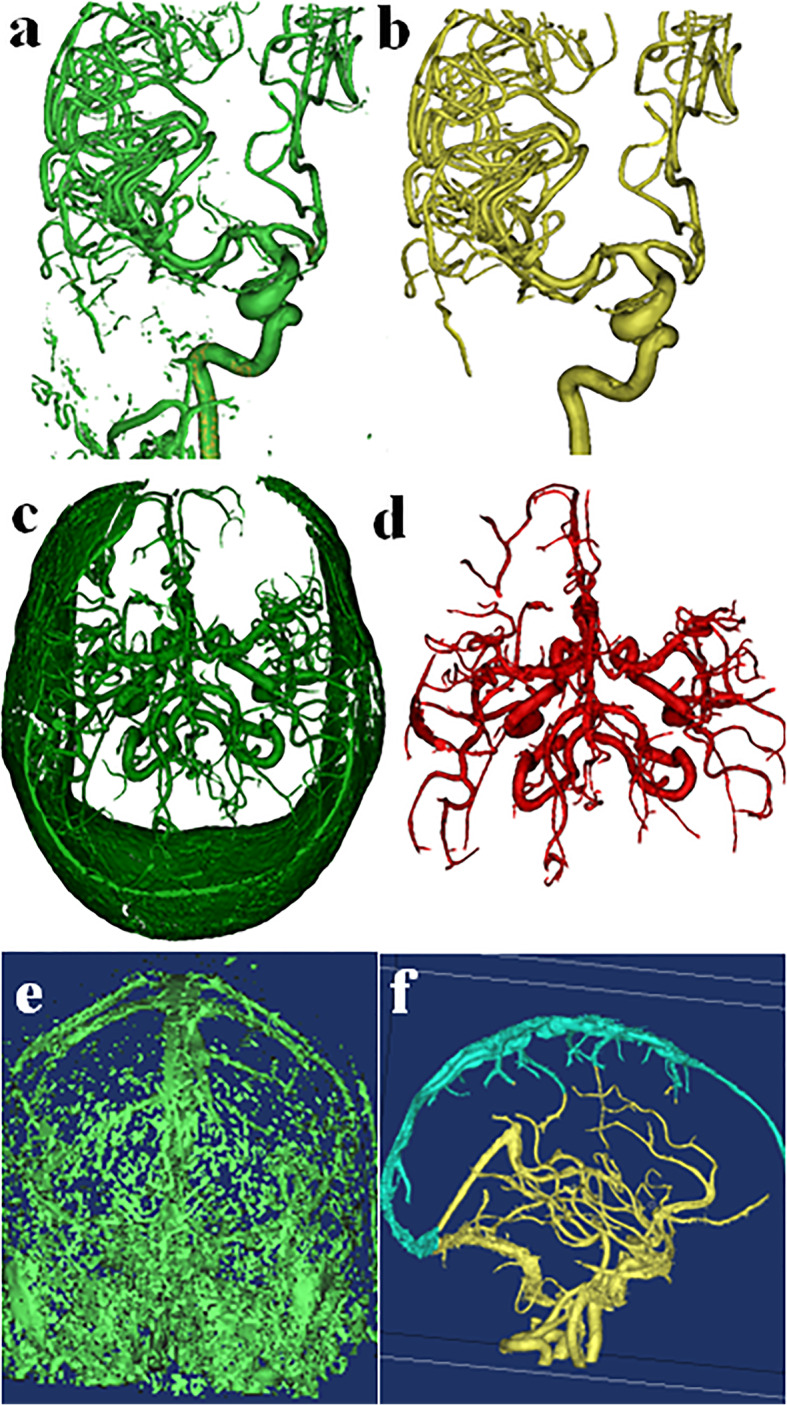
Segmentation of 3DRA, CTA, and MRA. **a** Initial 3D volume from thresholding a right carotid injection 3DRA includes only the vasculature due to the density of contrast and spatial resolution of angiography. **b** Region growing removes any external branches in one mouse click. 3DRA results in the most accurate 3D model of the intracranial vasculature in the fastest time. **c** Initial 3D volume from thresholding an MRA includes non-arterial structures to be removed. **d** Region growing removes any soft tissue and venous contamination in one mouse click, producing the second fastest and second most accurate vascular model with less robust distal vasculature than 3DRA. **e** CTA initial threshold shows that the arterial tree cannot be separated from bone, venous contamination, and some soft tissue by Hounsfield units alone. **f** After region growing, non-arterial structures, such as the dural venous sinuses (superior sagittal sinus, shaded light blue), remain that must be removed by manual trimming tools

In general, the fastest acquisition to segment is 3DRA, which takes < 5 min on average for an experienced user. Quick segmentation of 3DRA data is due to high contrast and spatial resolution, allowing thresholding and region growing to essentially eliminate all non-arterial background structures (Fig. 2a-b).

The second fastest acquisition to segment is TOF MRA, which takes less than 10 min. Similar to 3DRA, thresholding and region growing can eliminate nearly all non-arterial structures, and the primary arteries are easily segmented (Fig. 2c-d). However, small vessels may appear discontinuous and be difficult to segment. Additionally, artifacts such as pulsation, metal susceptibility, and motion may preclude accurate segmentation.

The most labor intensive of the acquisition techniques to segment, CTA, often takes longer than 30 min. Thresholding and region growing are usually of limited use due to overlapping density of bone and partially opacified veins adjacent to the arteries, even if bolus timing is optimal (Fig. 2e-f). Streak artifact from embolization material, aneurysm coils/clips, or dental amalgam may also have signal intensity similar to that of contrast-enhanced vessels, obscuring segmentation. As a result, user involvement in thresholding CTA is high, often requiring manually editing the 3D volume. For example, the skull base portion of a volume may need to be separately segmented, manually removing adjacent bone and veins. Dual energy acquisitions that allow for subtraction of the bone may aide in segmentation of the vasculature. However, errors, such as not removing all of the bone or removing a contrast-enhanced vessel, may occur, and stenosis adjacent to bone may be overestimated [18]. In the future artificial intelligence may be implemented to improve the segmentation process and decrease the amount of user time [19].

Finally, there are a number of software packages within radiology departments that may be used to segment the intracranial vasculature for the purpose of patient-specific 3D printing, such as Terarecon (Durham, NC), Phillips Intellispace Portal (Andover, MA), Siemens syngo.via (Malvern, PA), GE Healthcare AW server (Waukesha, WI), and Canon Vital Images (Minnetonka, MN). These are not currently Food and Drug Administration (FDA) -approved to export a Standard Tessellation Language (STL) file for the purpose of 3D printing a diagnostic model. The FDA does not regulate point-of-care manufacturing. However, it has issued guidance documents about the software that should be used [20]. At our institution we have chosen to use the FDA-approved software Materialise Mimics (Leuven, Belgium) for 3D printing patient-specific diagnostic models.

### Processing and creation of an STL file

**Fig. 3:**
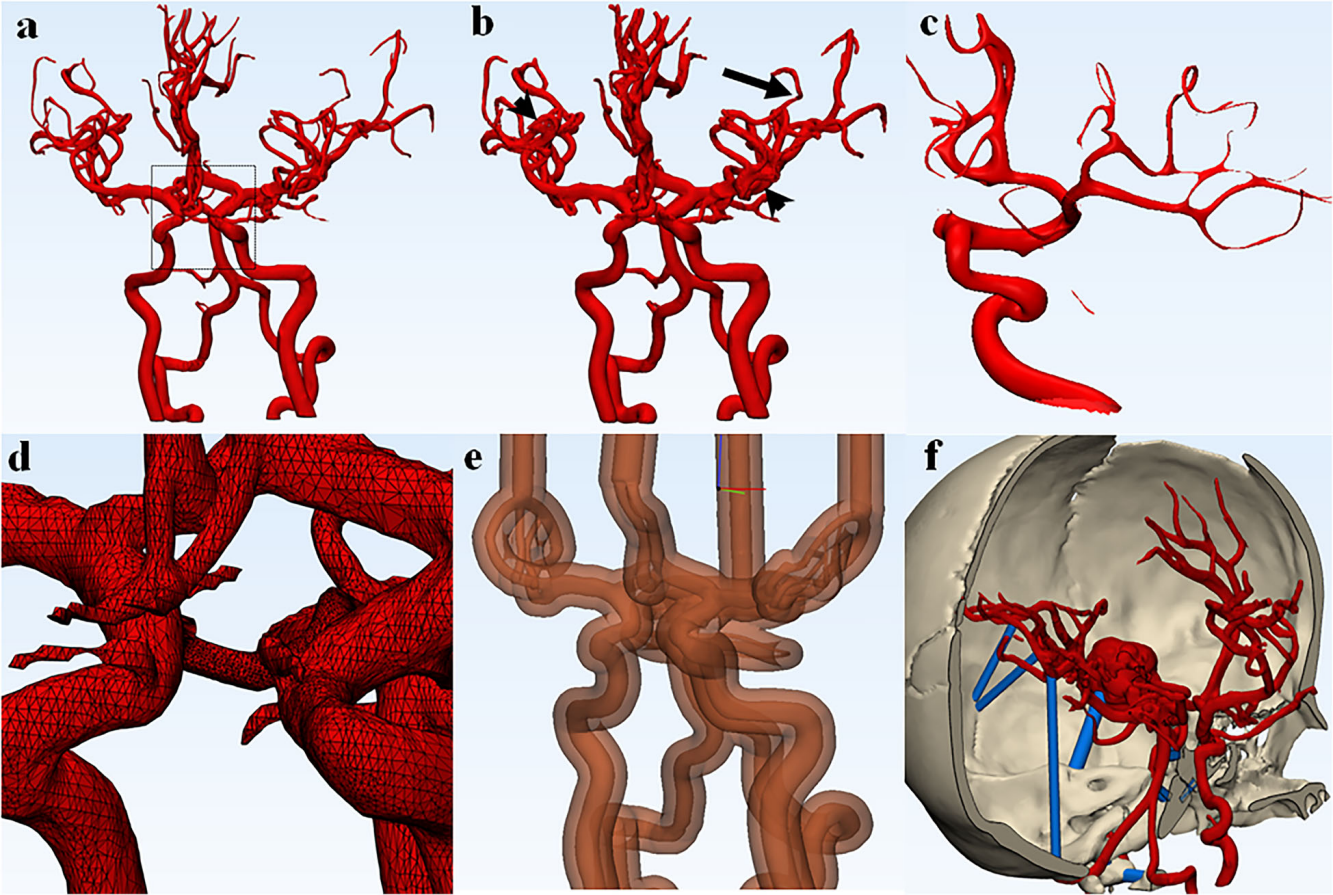
Examples of processing steps in CAD software. **a** Original segmented data set from a 3 T MRA. **b** Original data set can be wrapped to reinforce small vessels, (arrow) but this may result in merging or overlapping of vessels and loss of detail (arrowheads). **c** Example of over-smoothing. Smoothing creates a more realistic model removing triangulation from the mesh, though over-smoothing can result in attenuation or loss of small distal vessels and distortion of the anatomy. **d** Triangular mesh. 3D file is made of hundreds of thousands of triangles. Before printing, the mesh often has to be fixed to remove overlapping triangles, bad edges, and inverted normals. **e** Hollowed models used for patient-specific simulation or intracranial device research need to be modified in CAD software to create common outflow channels and lofted parts that can be assembled with physiologic pump systems. **f** Cylinders (blue) may need to be placed to support fragile anatomy so that parts that do not physically touch may be 3D printed to maintain anatomical relationships

Next, the segmented model is exported to CAD software (Materialise 3-Matic, Leuven, Belgium, as noted above for our institution), which creates mesh representations of the individual parts or volumes to be printed (Fig. 3). Within the software, post-processing steps of fixing, wrapping, and smoothing of the model are performed, as well as co-registration of multiple model parts, so that the CAD model is in a printable state.

Fixing the volume involves correcting flaws in the model, such as inverted triangles, overlapping triangles, bad edges, and noise shells [21]. In the intracranial vasculature this includes correcting artefactual discontinuities in vessels or removing small vessels that cannot be accurately printed.

Wrapping (Fig. 3b) puts a thin wrapper around the object of a specified thickness, filling anatomic holes to create a solid model without changing the anatomy. If this layer is thick, it may falsely increase the size of the vessels, which are on the order of a few mm. Of note, some prior studies creating hollow models for study of hemodynamics have dilated the vessels to account for the wall thickness of the printed model, aiming to replicate the true vessel lumen [2]. One must consider whether the thickness of the printed vessel will be built out or in from the segmented lumen, and whether the outer diameter or inner diameter of the vessel is desired to be closest to truth.

Smoothing (Fig. 3c) makes the model less triangular and more realistic. Without smoothing, vessels may appear to have triangular edges. However, over-smoothing may change the data and result in narrowing of the distal vessels.

Co-registration of multiple parts, such as vascular segments from separate DSA runs, vasculature relative to the skull, or vasculature to tumor, can be performed in the CAD software. However, current software does not provide automatic co-registration across exams or modalities, and it is challenging to co-register modalities that do not provide accurate representation of similar structures. For example, it is often helpful to print the vascular model with respect to the skull for surgical planning. However, the vasculature is best segmented from a 3DRA or MRA, while the skull is best segmented from a CT. To co-register the vasculature from an MRA and the skull from a CT, the skull must be segmented from CT, as well as from an MR brain sequence performed in conjunction with the MRA. The segmented MR and CT skulls are co-registered, and then the CAD program may register the MRA to the MR skull, and therefore, the CT skull as well. Finally, the MR skull piece may be removed. To perform these co-registrations, we us N-point registration methods in which three points of registration are manually placed on each CAD file; the best results occur when the points are placed as far as feasible from one another. The co-registration is manually adjusted, if needed, in the segmentation or CAD program using rotation and translation tools until the contours of the DICOM image line up.

To limit unnecessary expense of printing material and time, the model should be cut down to the smallest necessary component. Further, cuts can be made in the model if pieces are to be taken apart. Parts needed for stability may be designed.

Finally, the model is converted into a file format for printing, most commonly an STL file. These files define the model’s geometry but cannot be used to define other object properties, such as color, material, or texture. The geometry is defined by tessellation, using triangular surfaces to create the 3D surface (Fig. 3d). The smaller the individual triangular components are, the higher the level of detail and more realistic the anatomy, but also the larger the file size. Alternative formats include virtual reality modeling language (VRML) and object files (OBJ). The VRML files allow color to be defined in the model; the OBJ file format allows color, material, and texture to be defined. In addition to tessellation with polygons, free-form curves can be used to create surfaces. More precise models may be created with the OBJ file format; however, it is much more complex than STL.

### 3D printing and post-processing

**Fig. 4:**
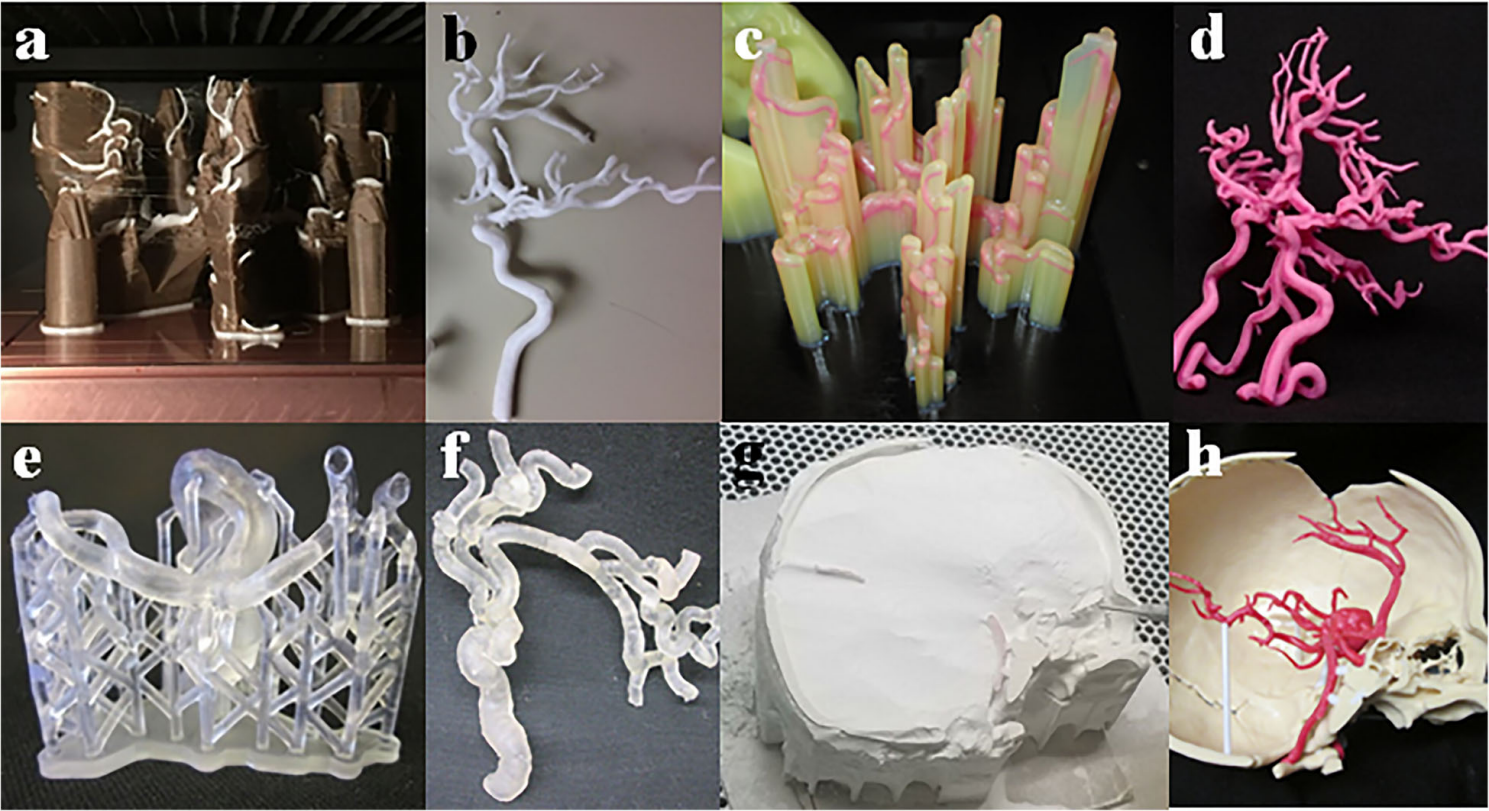
Examples of different 3D printing techniques and the required support structures. **a**-**b** Material extrusion, FDM (Stratasys Fortus, Eden Prairie, MN) model of the intracranial vasculature. **a** Intracranial vasculature with a water-soluble support structure. **b** The final model can be printed in only 1–2 materials and 1–2 colors. Resolution and fragility of printable models are dependent on the printer manufacturer and allowable materials. **c**-**d** Material jetting (Stratasys Objet 500 Polyjet, Eden Prairie, MN) model of the intracranial vasculature. **c** A model with surrounding soluble support material that can be removed by placement in a lye or water bath with ultrasonic agitation. **d** Final model printed in color. Material jetting allows full color multilateral printing. **e**-**f** Vat polymerization: SLA (Formlabs Form 2, Boston, MA) model of a posterior communicating aneurysm. **e** Vascular model with scaffolding support material is standard in this technology and can limit the ability to print complex internal architecture of hollow parts. **f** Final model with support removed. While flexible materials are available, they do not withstand physiologic pressure for device testing or simulation. **g**-**h** Binder jetting (3D Systems Projet 660, Eden Prairie, MN) model of an intracranial aneurysm printed in relation to the skull. **g** Support material is powder surrounding the print which is easily vacuumed and brushed away, saving significant post-processing time. **h** Final multicolor model is impregnated with cyanoacrylate to improve durability

Multiple 3D printing techniques are available, including vat photopolymerization, material extrusion, powder bed infusion, material jetting, binder jetting, direct energy deposition, and sheet lamination (Fig. 4) [9, 22, 23]. Direct energy deposition and sheet lamination are not suitable techniques for printing intracranial vasculature and will not be discussed further. Items to consider in choosing a printing method include level of obtainable detail and accuracy, available materials and fragility of the model, necessary support structure, post-processing/removal of support structure, available colors, ability to sterilize, temperature and moisture resistance, and time required to print. Primary challenges to printing the intracranial vasculature include printing life-sized small structures, removing support material without breaking the model, and achieving a hollow lumen, if necessary. If the model will be used for training, some materials are better for simulating osteotomies or craniotomies, while other materials are more durable for repeated endovascular training sessions. If the model is to be used for endovascular training, it may be beneficial to be translucent, but only certain technologies can print clear and flexible model parts. Printing and respective post-processing techniques are reviewed below with consideration of their use for creating an intracranial vasculature model. Available materials, average print cost, print and post-processing times for multiple print technologies are summarized in Table 2.

**Table 2:** Summary of printer technologies, average cost, print time, post-processing time, print layer thickness and available materials for printing one side of the anterior circulation, similar to as shown in Fig. 2b

Printer	Cost	Print Time (hr:min)	Post process Time (min)	Print Layer (mm)	Material for calculated cost	Other materials	Details
Material extrusion (FDM): Ultimaker	$26.98	42:39	24 Hours	0.06	Tough PLA and PVA	ABS, CPE, TPU, PVA, Nylon, PETG, Ryno, PET, HIPS	Dual material printer. Values given for highest resolution setting
Material extrusion (FDM): Prusa	$32.66	29:40	24 Hours	0.15	Tough PLA and PVA	ABS, CPE, TPU, PVA, Nylon, PETG, Ryno, PET, HIPS	May use four colors and a support. Values given for highest resolution setting
Vat polymerization (DLP): Newpro	$10.11	2:57	6 Hours	0.1	RG35	Veriguide OS, and Red Elastic	Single material printer
Vat polymerization (SLA): Formlabs	$7.52	15:52	6 h	0.05	Clear	White, Flexible, Elastic, Dental SG, Surgical guide, Denture LP, OP, RP, DP, Denture A1, A2, A3, A3.5, B1, B2	Single material cartridge
Powder bed fusion (SLS): HP	$80.00	7:57	45 Minutes	0.08	Nylon12, CYMK agents, fusing agent, detailing agent, and brightening agent		Uses all elements to color and strengthen the parts
Material jetting: Objet	$ 88.95	10:00	24 Hours	0.3	Vero Whiteplus and support 705	Vero Clear, Vero Agilus, Vero Cyan, Vero Magenta, Vero Yellow	May use three colors and a support
Binder jetting: Projet	$41.16	3:03	5 Hours	0.1	CYMK Agents, Core (Gypsum Powder), cleaning	Cyanoacrylate	Uses all elements to color and strengthen the part

*FDM* Fused deposition modeling, *DLP* Digital light processing, *SLA* Stereolithography, *SLS* Selective Laser Sintering, *PLA* Polysctic acid, *ABS* Acrylonitrile butadiene styrene, *PVA* Polyvinyl alcohol, *PETG* Glycolized polyester, *PET* Polyethylene terephthalate, *CPE* Co-polyester, *TPU* Thermoplastic polyurethane, *HIPS* High impact polystyrene

#### Material extrusion

Fused deposition modeling (FDM), the primary material extrusion process, uses a heated nozzle to extrude thermoplastics and create successive object layers (Fig. 4a-b). A dissolvable support material may be extruded out of a second nozzle and removed without damaging small structures (vessels) by placing the model in a lye or water bath with agitation over the course of a day. Flexible models can be printed with FDM, and it is accurate down to ~ 250 um layer thickness, which is less accurate than that afforded by stereolithography (SLA). There are numerous filament materials on the market that range from high temperature, increased tensile strength, metallic impregnated, ceramic mimicking, and flexible materials that can be used to create functional parts instead of simple prototypes. Filaments, such as acrylonitrile-butadiene-styrene (ABS), may be used to create a solid vessel lumen, which is then coated with silicone to produce the wall [4, 7]. The inner ABS is dissolved away with acetone to leave a hollow lumen. Benefits of FDM are its wide availability, quick speed, and low printer and material costs.

#### Vat Photopolymerization

Vat photopolymerization (Fig. 4e-f) methods use a laser or other light source to selectively solidify successive object layers on the surface or base of a vat of liquid photopolymer. The most commonly used vat photopolymerization method is SLA, which uses a single point laser to solidify material. Post-processing may include a solvent rinse, manually removing excess build material (e.g. clipping scaffold), and UV-light curing or “baking” to harden the resin. One of the most accurate printing methods with layer thickness of 25–100 μm, SLA provides a smooth finish and may produce semi-flexible models. Additionally, it may provide the clearest models, which may be desired for printing the skull so that the intracranial vasculature is still visible for endovascular training. However, only one or two colors may be used. Further drawbacks of SLA are its need for support structures for overhangs and within tortuous lumens, post-processing to remove those support structures, and the relatively brittle end-product.

#### Powder bed fusion

Powder bed fusion techniques of selective laser sintering and electron beam melting use a laser, electron beam, or other heat source to selectively fuse successive powder layers (plastic, metal, ceramic, or glass) to form a solid. Advantages of this technique are lack of a support structure, relatively little post-processing, and model durability. Commonly implemented for medical device manufacturing, powder bed infusion typically creates models using a single material. This technology has been used to make metal casts for cerebral aneurysm molds in various materials [24], which may be used for flow analysis. Drawbacks of powder bed infusion include its decreased accuracy compared to other methods.

#### Material jetting

Material jetting (Fig. 4c-d) is a method that uses multiple print heads to spray liquid layers that are solidified by exposure to UV light. This method provides high accuracy, to ~ 15 um layer thickness, and a smooth finish while using multiple materials, colors, and clear prints all from the same printer. The result is the ability to print the skull in solid material, the vasculature in color or flexible material, and the brain in another color or material. Not surprisingly, this coincides with increased material and printer costs. Support structures are required for overhanging parts or complex models, though they may be printed with the model using soluble material, shortening the post-processing time and decreasing the likelihood of breaking small vessels in the process.

#### Binder jetting

Binder jetting (Fig. 4g-h) uses a print head to selectively spray a binder (glue) onto successive layers of gypsum or metal powder. Post-processing includes vacuuming/blowing off unbonded powder and infiltrating with cyanoacrylate, wax, or resin. No support structure is required, as unbonded powder provides support during printing. A wide variety of colors may be used, which is of particular advantage when printing the vasculature in relation to other structures, such as skull or tumor. In addition, print time is shorter than other multicolor printing options. The disadvantage of binder jetting is the fragility of the models, which may be improved by infiltration with elastomers to create a flexible model. This technology is used in our practice for low-cost multicolor surgical planning and patient education models.

### Quality control

When printing models for medical use, regular quality control (QC) must be perform to ensure model fidelity. At our institution we have robust QC methods for segmentations, STL files, and printed models [5]. The segmented model and STL file are placed back on the acquired images, looking at the contours in all three planes to make sure the anatomy presented is accurate to the images. Caliper testing on the model is performed to ensure that key anatomic areas measure the same as the CAD file. Quality control tests are completed per build with coupons, weekly and monthly, with phantoms that are scanned via CT and mapped to a CAD file using finite element analysis. All printers are tested quarterly to assure they are operating within an acceptable tolerance level. Prior studies have demonstrated the ability to produce anatomically accurate models of the intracranial vasculature, such as aneurysms [2] and hollow patient-specific vascular models [25], though segmentation and processing steps may artificially increase vessel thickness or aneurysm size, in some cases [26].

### Applications

**Fig. 5:**
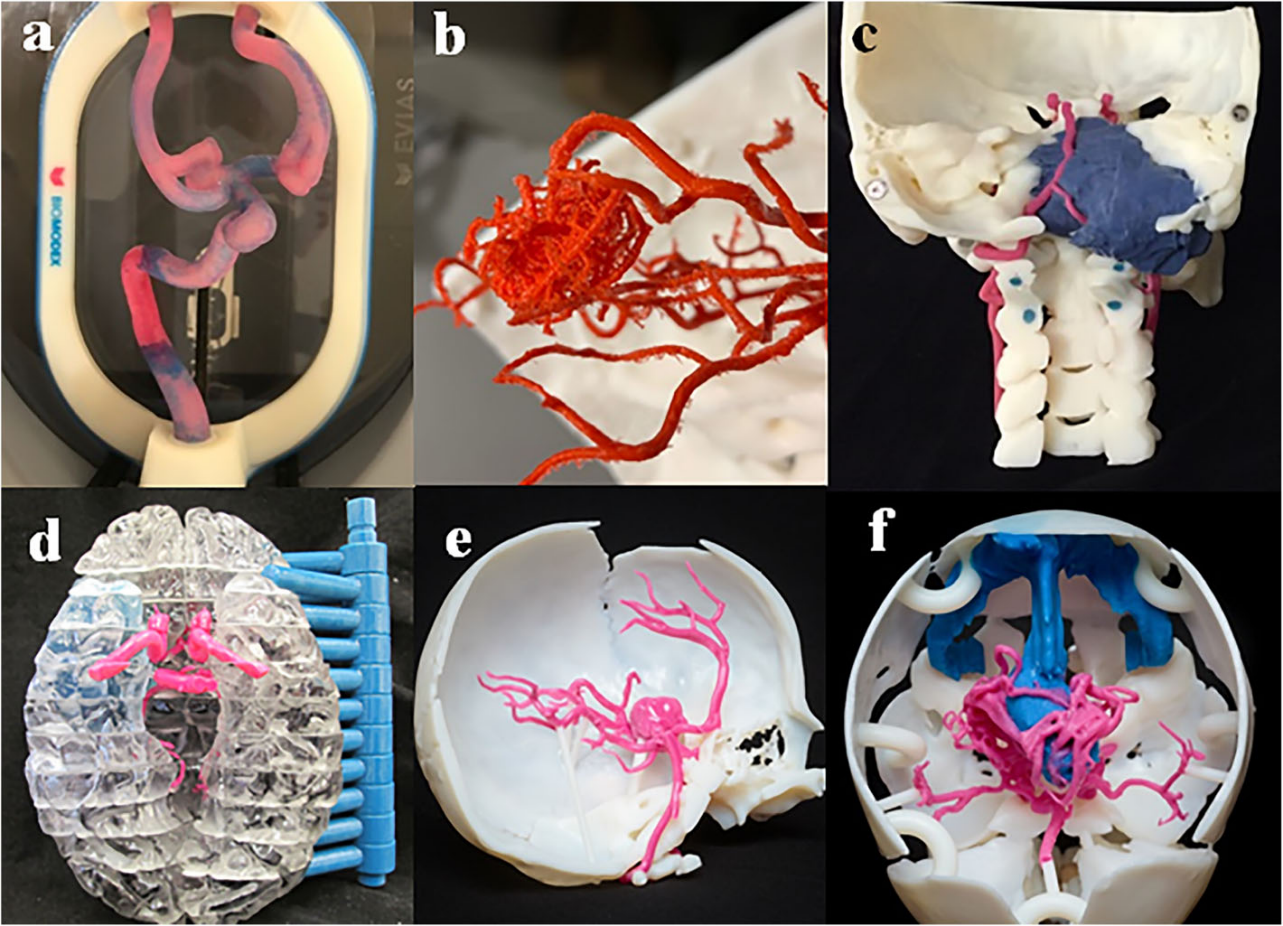
Clinical applications for 3D printing of the intracranial vasculature. **a** Hollow model in a patient with multiple intracranial aneurysms used for patient-specific simulation of aneurysm coiling (Biomodex, Paris, France). **b** Parasagittal arteriovenous malformation (FDM) printed in relation to the skull and used for patient education in addition to surgical planning. **c** Skull base tumor (Material jetting, Objet 500). The surgical approach was changed after seeing the model with the detachable posterior component. **d** Insular glioma (Material jetting, Objet 500) in which 3D printing was used to demonstrate the relationship of the intracranial vasculature to critical structures. **e** Intracranial aneurysm printed in relation to the skull in a 7-year-old (Material jetting, Objet 500), and (**f**) vein of Galen malformation printed in relation to the skull (Material jetting, Objet 500) in a 6 month old. Both e and f are patient-specific models of rare pathology used for pre-surgical planning and ongoing Accreditation Council for Graduate Medical Education radiology, neurosurgery, and neuropathology education. The model of the vein of Galen malformation (f) changed the treatment to a two stage approach of interventional therapy followed by surgical intervention from a single interventional treatment plan

Intracranial vasculature 3D printing has been applied for education, training, and pre-surgical planning (Fig. 5). In education, 3D models improve understanding of complex vascular anatomy or anatomic variants for patients, trainees, non-specialty staff, and practicing neurosurgeons [3, 4, 27–29]. The 3D model not only allows for visualization of structures in three dimensions, but also the ability to physically manipulate the model, which is not possible with visualization techniques like virtual reality or augmented reality.

In surgical training, 3D models of the intracranial vasculature have been widely used for surgical simulation of endovascular treatment [30, 31] and surgical clipping of aneurysms [32, 33]. Simulation has become increasingly important for trainees given the more complex cases being targeted for therapy and variety of treatment options. In particular, as endovascular treatment has become more widely implemented, fewer aneurysms are treated via surgical clipping, yet skills must be learned and maintained [4, 32].

Patient-specific models of the intracranial vasculature have been implemented for presurgical planning in cases of aneurysm coiling or clipping [4, 29, 30, 32] and tumor resection [27, 34]. The 3D-printed models have been shown to provide a high level of accuracy and assist in defining the relationship of the targeted lesion (aneurysm or tumor) to surrounding vasculature, brain, cranial nerves, and bone. Benefits of using patient-specific 3D models of the neurovasculature include improved selection of surgical candidates, improved planning of the surgical approach, decreased complications, and decreased operative time [28, 31, 34].

Although 3D printing could be applied for any intracranial vascular pathology, at our institution the focus has been on use of patient-specific models for patients with altered anatomy, congenital malformations, rare malformations, and complex cases. For cost efficiency, patient-specific models are not necessary for treatment planning of straightforward aneurysms. However, printing of simple cases is useful for medical device testing, medical device development, ACGME training, and patient-specific simulation for new staff to steepen learning curve and reduce fluoroscopy time.

### Future directions

As 3D printing in general becomes more commonly applied, the costs will need to be justified. For common pathologies the cost of intracranial vascular 3D printing may be justified via randomized control trials that demonstrate benefits of 3D printed models. However, for rare and complex pathologies, randomized control trials will not be feasible due to insufficient number of cases to reach statistical power.

## Conclusions

Printing of intracranial vasculature in 3D may be utilized for multiple applications, including patient-specific models, training, and education. For 3D printing to be implemented as a clinical service, the DSA, MRA, or CTA acquisition must be optimized to allow for accurate and efficient segmentation. In preparing the CAD files for printing the model, one must consider how each step in the process will affect the model’s accuracy and the ability to print. Finally, the appropriate printing technology and material must be chosen to best fit the desired application. In reviewing these processes, we provide insight into the manufacturing of 3D models of the intracranial vasculature that may facilitate incorporation into or improve utility of 3D vascular models in clinical practice.

## Data Availability

Not applicable.

## Abbreviations

3D: Three dimensional
3DRA: 3D rotational angiography
ABS: Acrylonitrile-butadiene-styrene
CAD: Computer-aided design
COW: Circle of willis
CT: Computed tomography
CTA: Computed tomography angiography
DICOM: Digital imaging and communications in medicine
DSA: Digital subtraction angiography
FDA: Food and drug administration
FDM: Fused deposition modeling
IV: Intravenous
MRA: Magnetic resonance angiography
OBJ: Object
QC: Quality control
SLA: Stereolithography
STL: Standard tessellation language
TOF: Time-of-flight
VRML: Virtual reality modeling language
